# Some Aspects of Association Aims and Work1Read before the Keystone Veterinary Medical Association, Philadelphia, September, 1896.

**Published:** 1897-08

**Authors:** W. Horace Hoskins

**Affiliations:** Philadelphia, Pa.


### THE JOURNAL
OF
### COMPARATIVE MEDICINE AND VETERINARY ARCHIVES.

VOL. XVIII. *AUGUST*, 1897. No. 8.

### SOME ASPECTS OF ASSOCIATION AIMS AND WORK.[1]

BY W. HORACE HOSKINS, D.V.S.,
PHILADELPHIA, PA.

MR. PRESIDENT, FELLOW-MEMBERS AND COLLEAGUES:

So often have I been heard upon some of the lines of thought that flow forth from Association privileges and blessings that it seems proper for me to offer you an apology for again craving your indulgence and attention as listeners for a few minutes to "Some Aspects of Association Aims and Work."

So much pleasure, advantage, and assistance have I ever found in my association privileges, and continually there seem to rise up aspects of greater value in association membership and fellowship, that I am led to wonder at times at the seeming indifference of some to the personal advantages of membership in these organizations, not to speak of their power for good in every community where they exist.

**Nashville, our Mecca for 1897.**

Never, perhaps, were they of so much importance to our welfare as at the present hour. Never was there need of such close unity of our members as now. Never before have conditions and changes so peculiar in our country's history culminated at a time when we were so ill-prepared to deal with them individually as at the present time. Never were the temptations so strong to gain personal advantage of one another, in the end of so little worth to the individual, and of such great danger to the general good. At no time in the history of our profession were such great opportunities for any body of men that ever lived so

[1] Read before the Keystone Veterinary Medical Association, Philadelphia, September, 1896.

promising of good results if united efforts, properly expended, for the common good of all be put forth. In times of prosperity the parasite upon the body politic seems only to do an insignificant amount of harm; in the days of adversity and trouble he multiplies, and the pathway of recovery from times of depression is long and trying and the results slow and unsatisfactory.

**Tennessee the 16th State to enter this glorious Union.**

We have watched the growth of great industries in our land and the multiplication of great industrial establishments with a pride and glory that seem to us now peculiar, and I feel that we have been vieing with one another in our admiration, forgetful of how far-reaching and dangerous these have been to our own welfare and prosperity. I shall cite simply for an instance one great establishment of our own city, a great mart of trade, toward which every channel of communication seems to run. Its influences have been felt (not for the general good) in every section of our great city and for a territory of one hundred and more miles from its central location. It has concentrated under its roof the business of fifty or more individual houses; it has robbed our community of the value of fifty or more individual men whose independence and wishes for the common good of all within our borders were ever outspoken in thought and heroic in action for every movement for the advancement of our general prosperity. We have vied with one another in carrying to this great mart of trade a goodly portion of our earnings, to the detriment of our neighbors and against public good for all. We have seen wiped out here the private horse-equipment establishments of fifty or more individual concerns. We have striven to outdo each other in securing the professional work of this large aggregation of horses controlled by one establishment, and we have noticed the respective professional bids of $1000, $625, and $250 for professional services and medicines to this single stable annually. We have seen the bid of $250 win, and as a result we have had a loss to the profession of not less than $1250 per annum, quite sufficient in itself to maintain a good, rising young member in our profession. But this has been only one side of the loss. The manufacturer of drugs and chemicals has suffered; the retailer and dispenser of medicines has suffered; the carriage-builder has met with a loss, for one carriage has supplanted what fifteen or twenty would have been engaged in, and the number of horses required among our members is lessened; the furnisher of food-products has suffered; the harnessmakers' income reduced; and the horseshoer has a less amount of work. What seemed a blessing in disguise is an evil and a menace to the community, an injury to public good, and prejudicial to our welfare as a whole. Look around you, fellow-members, for the picture I have drawn may be seen in every large community, and they continue to multiply on every side.

Now, where does this become a factor in an association aspect? Our bond of unity would not contribute to the increase of these institutions were we more thoroughly organized, and when from time to time we would discuss the necessary side of our work as to rates of remuneration, for every advantage gained by such institutions or corporations contributes to their multiplication in other directions, and the safety and prosperity of a whole people depend wholly upon the relative propserity of all the people who make up a community. Therefore, the greater the number of patrons that contribute to your sustenance and support is your greater security, the higher welfare of your profession, and your stronger independence as a man and citizen among your people. A few supporters make you more subservient to the wishes of a smaller number of people, among whom you will always find some who are selfishly inclined and whose influence you do not sympathize with, and who would make you a carrier of burdens that are not always easy to bear. Your profession can only advance in its true ratio when the common prosperity of all is the highest. A few of your number rapidly obtaining wealth and material prosperity means that the greater number are lingering in the throes of uncertain employment. Association power, united in its aims, will sooner solve such a condition for us than the sum total of individual theories left to determine the best remedy.

**Our first convention in the new South, at Nashville, Sept. 7th, 8th, and 9th.**

Association unity has solved the question of higher education and brought the weaker schools to a lively sense of the needs of the hour, and your future colleagues in our profession will not be but half-equipped to fulfil its duties, as you and I have been; and when grave periods of depression that have come twice to us as a profession in the history of our country and to the equine industry (I refer to the advent of steam and the death of the stage-coach and canal tow-path, the advent of electricity and the death of the horse-car) there will not be so many of us stranded because our education did not extend beyond the ills of the equine race, when a whole country suffers in every city, town, and hamlet for inspectors of animal food-products, and the insufficient number of men equipped for this work makes the agitation for its establishment over the land a comedy with a tragic side. Had associations fulfilled earlier one of their missions—to advance veterinary education at every educational post—there would be to-day greater prosperity for all, and the few schools that early sought to establish higher education would not have been handicapped by those who forgot quality in the zeal for quantity, but would have advanced still higher, and the great number of those in practice to-day would have been equipped to fill these pressing needs for our people, and their agitation for sanitary police more potent in results, more fruitful in returns than they have been. Association privileges must come now, freighted with the responsibility of being quick to recognize every step forward of every centre of learning in our land, and its strong arm of support extended to sustain them. We must gradually exact greater safeguards for our people from the standpoint of citizenship, in vouchsafing to them the character and learning of those who are to be intrusted with these positions of trust, and in whose hand and keeping are to be placed these evidences of material wealth and prosperity wrapped up in our livestock of this great country.

**Nashville, the scene of important events in our Nation's history.**

In every community we must take the initiative in leading every movement that comes within the domain of our work. We must direct the channels into which our labors shall flow, and to do this we must be united and first determine what is the best plan and what will bring the greatest good to the greatest number, and with a strong and united arm of strength lift our people up to the light of our own horizon, that they may see the good that is to flow from our plans and purposes. There is work for every member of our profession; there is something your fellow-colleague can do better than you, and by association fellowship you will best learn this, and be better able to direct with forceful results his efforts for the common good of all.

I see struggling over our State to-day in more than a score of cities and towns individual veterinarians fighting single-handed the battle for greater safeguards for their people in purer food-products. Some of them, I know, have never considered the value of association power and assistance; all of them to-day need it, and some of them are casting around, like fish out of water, for some support to lean up against, for some help to fall down upon them from somewhere—help and assistance that only come from association circles. This aspect of association work is broad and far-reaching, and I might go on and show you its multitude of ramifications, but I would tire you with details that I know are now filling your minds with food for thoughtful consideration, and which will come back to us in the future like bread thrown upon the waters, with a greater return than any I can picture now.

It was association power and its consideration in organized bodies of people banded together for the common good of all that has fastened the merit-system of appointment in our public service. It reached our own professional organizations, and its voice was not unheard by those charged with its needs in those positions of our public service requiring veterinarians; and it no doubt helped in some measure to strengthen and hasten the completion of the plans which now exist in our government veterinary service, where every member of our profession stands on equal footing and where promotion is made solely upon merit and ability. And no one rejoices more in this state of affairs to-day than the former appointing power—Secretary Morton—from whose pen I have had the words that it has enhanced the character of the service obtained by our government, and the system of work moves along in a thoroughly smooth and efficient manner.

**The new South, the centre of the greatest advancement of our country at the present time.**

It was the absence of strong association circles and fellowship that helped to perpetuate the infamous spoils-system so long. In a country where so intelligent a people live, and where once obliterated and the people have tasted of an honest and efficiently administered civil-service, they will never return again to the dangers and bondage of a system that was cruel and barbarous in the extreme. The absence of association fellowship and fraternal affiliation made it easy, and violated no pledge of good faith, no code of ethics, for John Smith to have removed William Brown, no matter the relative worth of each other, from the public service, but because there was a change in the head of government of our country, State, or city or that Smith had been more subservient to the political advancement of the appointing power, the good of the people, the unjust reflection and aspersion cast upon the profession of their adoption was of secondary consideration, and what ground was lost in the second sober thought of the great majority of the people in their respective communities, not measured until it was too late to stay its injurious influences, and for which the great body of us must struggle to recover. Thus, another aspect of association aims and purposes is mirrored before you; its ramifications and influences I leave for you to trace and better appreciate than I can conjecture them, for some of you have tasted of political appointments and felt the retributive blows and hardship of such places under the pernicious spoils-system, that can never win approval from association circles, or be a concession of ethical consideration when framing rules of honor for our welfare and general good, and for which only the punishment of loss of association privileges and the esteem of our colleagues stands as the dire sentence for its betrayal—a punishment so potent and forceful that I have witnessed it rob the manhood and self-respect of some members of our profession, that they have expressed the wish that they had never entered the calling, some of whom have dropped by the wayside, and are only lingering on for a time at the outposts of the profession, without the fellowship of their colleagues, the esteem of their community, and who are strangers to every advance of their profession, and factors only for good in a feeble way in any direction.

**In Tennessee are the most noted stock-raising farms in America.**

These are some of the results of lack of association fellowship and unity, and they will become no less potent in their unhappy side as the advance of our calling moves on with greater strides, for I see in the future the great body of our people seeking foremost channels through which they can help the general advancement of all.

Permit me to add one more purpose of association unity and I will close my subject. It is that of settling all our differences of opinion on questions of public import within our organization. It is to present more forcibly the value of asscoiation unity, where our final opinion on all subjects of importance to us as a profession is the sum total of the best thoughts and plans of all combined; it is the burnished and refined, the polished and brightened single reflection of associated rays, that have shot forth from a multitude of sources, and when so presented has added strength and force that win for it support and approval and make it potent for good. I can but add to this point an illustration more forceful than words, and of which some of my hearers may recognize the source of my pen picture. The scene of action—a great commonwealth honored by strong men in our calling. A great sanitary police project is agitated, the people are aroused, the profession are at a diversity of opinion. The magnitude of the undertaking is seemingly understood but by few; the need and value of its accomplishment acknowledged by all. The plans to be pursued must of necessity be original; but there are those of our number who have studied it out. The powers of the State, while startled at the undertaking, are reliant upon the projectors, and their confidence is displayed by their concession of the power to carry the project into execution. The profession of the State are not united; there are many men of many minds; they cannot compass the great good for all if there be not unity of thought and action. They do not grasp the great opportunity; they assemble in partial strength; they discuss the movement not to arrive at a single plan of action, but to differ for reasons that seemed to be not wholly unselfish in character. There must be leaders, and those best equipped seem to vary in the opinion of those assembled; the sessions are stormy, the division of opinion grows stronger; the agitation of methods and plans are carried on beyond the confines of professional circles; the layman enters the field and adds to the difficulties, "for fools rush in where angels fear to tread;" the association's influence is disrupted, the value of its opinion lost; the worth of its existence greatly shattered, friendships and fellowships severed, and chaos prevails. The laymen propose to settle the scientific aspect of the situation; the professional men the business aspect, and the whole commonwealth is agitated as never before. The value of the work is discounted; the methods to be pursued are thrown back for laymen to decide; the whole civilized world is threatened with heavy loss, though an able community, an advanced people have elected to undertake a work of world-wide magnitude and of importance to every man, woman, and child of the civilized and uncivilized world. But the work was must go on; leaders have arisen at every need and demand of the world's progress, and, though tried and persecuted, they push on, under difficulties and trials little appreciated by those not wholly familiar with the great battle. The work is impeded, the plans are changed; but, with determination, every foot of the ground is fought over, and day by day is revealed the but faintly estimated value of the work done and the work needful for all.

**The centennial of her admission to the roll of States.**

How much has been lost can never be estimated or realized, and how serious a responsibility will rest upon those who would impede the progress, time only will tell. For my purpose, suffice it to say that the work of a valued association has been nearly lost to us all for more than a year; its ulterior influences have been felt in our national organization, and it will require years of unremitting labor to regain that which is lost, and which loss and burden should never have been made or imposed upon a profession whose burdens are many and whose yoke is not easy to bear.

Such, then, is another aspect of association aims and purposes; and its further thought and consideration I leave with you.

